# A Follow-Up of the Multicenter Collaborative Study on HIV-1 Drug Resistance and Tropism Testing Using 454 Ultra Deep Pyrosequencing

**DOI:** 10.1371/journal.pone.0146687

**Published:** 2016-01-12

**Authors:** Elizabeth P. St. John, Birgitte B. Simen, Gregory S. Turenchalk, Michael S. Braverman, Isabella Abbate, Jeroen Aerssens, Olivier Bouchez, Christian Gabriel, Jacques Izopet, Karolin Meixenberger, Francesca Di Giallonardo, Ralph Schlapbach, Roger Paredes, James Sakwa, Gudrun G. Schmitz-Agheguian, Alexander Thielen, Martin Victor, Karin J. Metzner, Martin P. Däumer, 454 HIV-1 Alpha Study Group

**Affiliations:** 1 454 Life Sciences, A Roche Company, Branford, CT, United States of America; 2 National Institute for Infectious Diseases “L. Spallanzani, Rome, Italy; 3 Janssen Infectious Diseases—Diagnostics bvba, Beerse, Belgium; 4 Plateforme Génomique Toulouse/Laboratoire Génétique Cellulaire, Toulouse, France; 5 Blutzentrale Linz, Linz, Austria; 6 INSERM U563, Toulouse, France; 7 Robert Koch-Institute, Berlin, Germany; 8 Division of Infectious Diseases and Hospital Epidemiology, University Hospital Zurich, University of Zurich, Zurich, Switzerland; 9 Functional Genomics Center Zurich, University of Zurich, ETH Zurich, Zurich, Switzerland; 10 Institut de Recerca de la SIDA–IrsiCaixa, Badalona, Spain; 11 Technology Innovation Agency-National Genomics Platform, Durban, South Africa; 12 Roche Applied Science, Penzberg, Germany; 13 Institute of Immunology and Genetics, Kaiserslautern, Germany; 14 Institute of Medical Virology, University of Zurich, Zurich, Switzerland; University of Pittsburgh, UNITED STATES

## Abstract

**Background:**

Ultra deep sequencing is of increasing use not only in research but also in diagnostics. For implementation of ultra deep sequencing assays in clinical laboratories for routine diagnostics, intra- and inter-laboratory testing are of the utmost importance.

**Methods:**

A multicenter study was conducted to validate an updated assay design for 454 Life Sciences’ GS FLX Titanium system targeting protease/reverse transcriptase (RTP) and *env* (V3) regions to identify HIV-1 drug-resistance mutations and determine co-receptor use with high sensitivity. The study included 30 HIV-1 subtype B and 6 subtype non-B samples with viral titers (VT) of 3,940–447,400 copies/mL, two dilution series (52,129–1,340 and 25,130–734 copies/mL), and triplicate samples. Amplicons spanning PR codons 10–99, RT codons 1–251 and the entire V3 region were generated using barcoded primers. Analysis was performed using the GS Amplicon Variant Analyzer and geno2pheno for tropism. For comparison, population sequencing was performed using the ViroSeq HIV-1 genotyping system.

**Results:**

The median sequencing depth across the 11 sites was 1,829 reads per position for RTP (IQR 592–3,488) and 2,410 for V3 (IQR 786–3,695). 10 preselected drug resistant variants were measured across sites and showed high inter-laboratory correlation across all sites with data (P<0.001). The triplicate samples of a plasmid mixture confirmed the high inter-laboratory consistency (mean% ± stdev: 4.6 ±0.5, 4.8 ±0.4, 4.9 ±0.3) and revealed good intra-laboratory consistency (mean% range ± stdev range: 4.2–5.2 ± 0.04–0.65). In the two dilutions series, no variants >20% were missed, variants 2–10% were detected at most sites (even at low VT), and variants 1–2% were detected by some sites. All mutations detected by population sequencing were also detected by UDS.

**Conclusions:**

This assay design results in an accurate and reproducible approach to analyze HIV-1 mutant spectra, even at variant frequencies well below those routinely detectable by population sequencing.

## Introduction

HIV-1 drug resistance testing is currently recommended and widely implemented as the standard of care prior to starting antiretroviral therapy (ART) [1]. The standard method used worldwide for drug resistance testing is based on population sequencing, which does not allow for the detection or reporting of minority HIV-1 drug-resistance mutations [2]. Several methods are available for detection of minority mutations [3]. Tests based on allele specific PCR (AS-PCR) can be used for high sensitivity detection of minority variants; however, this technology can only detect one mutation per assay and becomes very labor intensive for analysis of multiple resistant variants [4, 5]. Next Generation Sequencing assays, having the capability to analyze multiple drug resistance mutations in a single assay, are being used with increasing frequency [6]. Ultra deep sequencing (UDS) has been retrospectively assessed in clinical trials such as the MOTIVATE and A4001029 studies where viral tropism was determined phenotypically prior to maraviroc administration [7]. The presence of ≥2% non-R5 HIV by UDS was found to be a good predictor of virologic response to maraviroc [7].

UDS has been indicated in previous studies to be an important tool for drug resistance detection due to its ability to detect and quantify minority drug resistance mutations. A systematic review and pooled analysis showed the risk of virological failure to be significantly increased in ART-naïve patients receiving an NNRTI-based first line regimen who harbor pre-existing minority NNRTI-resistant HIV-1 variants [8] recently confirmed by a multi-cohort European case-control study [9]. The latter and all of the studies analyzed in this pooled analysis; however, were retrospective in nature. Prospective studies have not yet been completed to unequivocally show the clinical relevance of minority HIV-1 drug-resistance mutations. Furthermore, clinical cut-offs for the significance of such minority HIV-1 drug-resistance mutations have yet to be defined. More sensitive assays and studies are required to answer these questions. Here, we describe an assay with the potential to help answer these questions.

We previously described a multicenter study demonstrating high inter-laboratory correlation of UDS-detected minority HIV-1 drug-resistance mutations exclusively in HIV-1 subtype B samples. This initial study was completed using the GS FLX sequencing platform [10] and focused on the detection of minority HIV-1 drug-resistance mutations in the RTP region of HIV-1 subtype B viruses. The follow-up study described here employed an updated primer design consisting of 8 amplicons between 400 and 600 bp (including sequencing adaptors and multiplex identifier (MID); also commonly referred to as a barcode) that target multiple clades of HIV-1 samples. The new assay design takes advantage of longer read lengths and also includes the V3 loop of the *env* gene to provide viral co-receptor use information (Fig A in S1 File).

Validation based on comparison to state of the art genotyping is a pre-requisite for adoption of ultra deep sequencing in routine care settings. We investigated the inter- and intra-laboratory performance of a prototype HIV-1 primer set and sequencing protocol for ultra-deep pyrosequencing. In addition, we sought to demonstrate concordance with state of the art genotyping as well as the detection of minority HIV-1 drug-resistance mutations in samples from multiple HIV-1 clades.

## Materials and Methods

### Ethics statement

Human plasma used for this study was derived from anonymous blood donations obtained from healthy blood donors provided by the Blood Donation Service. Written informed consent or an ethics statement for the use of plasma from anonymous blood donations are not required for research purposes.

### Study design

HIV-1 containing samples were provided to 10 study sites in Europe and one study site in South Africa. Site numbering began with the number 20 to disambiguate the new data files from those generated in the previous study. The samples were comprised of 30 HIV-1 subtype B and 6 subtype non-B samples (A2, AE, A/G, B/G, C, and G) with viral titers of 3,940–447,400 copies/mL, two dilution series (52,129–1,340 and 25,130–734 copies/mL, respectively), triplicate samples and two plasmid controls (Table A in S1 File). All samples were sent blinded to the sites. Also provided to each site were detailed laboratory protocols, four 96-well thermocycler plates with ready to use cDNA primers as well a set of four 96-well thermocycler plates that contained pre-loaded barcoded PCR primers for the generation of eight overlapping amplicons. For traceability through the workflow, the barcode on the PCR primers in the first column of each plate was unique to that plate. For the concordance analysis, population sequencing of all samples was performed with the ViroSeq HIV-1 genotyping system by Dr. Däumer’s clinical laboratory.

### Primer design

The overlapping amplicons span PR codons 10–99, RT codons 1–251 and the entire V3 region. The amplicons were designed to provide dual coverage for Stanford Database (version 6.0.9) Drug Resistance PR and RT mutations with a score ≥5 (any drug) and tropism determinants in V3 (Fig A and Table A in S1 File). All primers were designed using the principles previously described, with the exception of allowing for 98% conservations instead of 99% as described in the referenced paper [11]. The longer read length chemistry employed in this study reduced the required number of overlapping RTP amplicons from six to four and also allowed for placement of primers in sufficiently conserved areas outside the V3 region.

### Sample characteristics

The RTP samples used in this study were cultured recombinant virus diluted in plasma from HIV-1 negative individuals. The cloned inserts included the coding regions for the complete protease and the 5’ 302 amino acids (aa) of the reverse transcriptase (nucleotide positions 2,002 to 3,457 based on the reference sequence HIV-1_HXB2_, GenBank accession number K03455). The V3 samples were cultured samples from patient isolates, diluted in HIV-1 negative plasma. Two control samples were generated for inter- and intra-laboratory comparison purposes (control sample A) and for amplification control (control sample B). Control sample A is a mixture of two plasmid samples obtained through the AIDS Research and Reference Reagent Program, Division of AIDS, NIAID, NIH: pM46I/L63P/V82T/I84V and pL10R/M46I/L63P/V82T/I84V from Dr. Emilio Emini [12]. These two plasmids were mixed at a 20:1 ratio to create a sample with the L10R mutation at approximately 5%. Control sample B is a preparation of plasmid pM46I/L63P/V82T/I84V.

### HIV-1 RNA extraction, cDNA synthesis, amplification and pooling

1 ml of each sample was ultracentrifuged for 1.5 hrs at 4°C. 800 μl of the supernatant was removed and discarded, leaving ~200 μL of viral pellet and plasma. HIV-1 RNA was extracted using the QIAamp MinElute Virus Spin kit (Qiagen, Hilden, Germany) according to the manufacturer’s instructions. MS2 carrier RNA (Roche, Mannheim, Germany) was then added to each eluate to a final concentration of 10 ng/μL. 13 μL of the RNA eluate was reverse transcribed using Transcriptor Reverse Transcriptase (Roche) with each of the “4R”, “5R” and/or “V3R” primers (provided in ready to use 96-well plates), resulting in three first strand cDNA-fragments covering the protease, reverse transcriptase and *env* V3 coding regions of HIV-1. The cDNA was treated with RNAseH (Roche) and 3 μL cDNA product used for each PCR reaction.

Amplicons were generated from the cDNA and the two plasmid control samples (A and B) by 40 cycles of PCR with FastStart HiFi Polymerase (Roche) as depicted in Fig A in S1 File using PCR conditions previously described [10] in a total of four 96-well thermocycler plates. Primer sequences are given in Table B in S1 File. Amplicons were then purified with AMPure XP beads (Agencourt, Beckman Coulter Inc.) and quantified using the Quant-iT PicoGreen dsDNA kit (Invitrogen, Darmstadt, Germany). Amplicons with concentrations <5 ng/μL were further analyzed on a 2100 Bioanalyzer (Agilent Technologies, Böblingen, Germany). PCR products with a molar ratio of primer-dimer to amplicon above 1:3 were excluded from sample pooling. The remaining amplicons were pooled equimolarly by sample MID (6 RTP and 2 V3 amplicons for each MID), with any missing amplicon being compensated for by increasing the amount of an overlapping amplicon, where possible. The samples were then pooled by plate to make 4 final pools, 1 pool per plate corresponding to 1 pool per region of a 454 sequencing pico titer plate (PTP) fitted with a 4-lane gasket. In total, 36 samples (18 for sequencing of the RTP region and 18 for the env V3 region) were amplified in PCR plates 1 and 2, corresponding to regions 1 and 2 of the sequencing run. 3 additional samples were amplified in triplicate on plate 3 (9 samples total, sequencing both the RTP- and env V3 regions), corresponding to region 3 of the sequencing run. Amplicons for the two sample dilution series were generated on plate 4 (10 samples total, sequencing both the RTP- and env V3 regions), corresponding to region 4. Control sample A was amplified in each of the first 3 plates and included in the pools for sequencing. Control sample B was used as an amplification control on each of the four plates to ensure proper execution of the PCR step as was a negative water control to ensure no contamination of the PCR; neither of these controls was included in the pools for sequencing.

### Ultra deep sequencing

Each of the 4 amplicon pools was separately subjected to clonal emulsion PCR amplification on beads using reagents that enabled sequencing in both the forward and reverse directions (Lib-A kit, 454 Life Sciences—A Roche Company, Branford). DNA-containing beads were counted on a Multisizer3 instrument (Beckman Coulter Inc.) and prepared for sequencing. Approximately 790,000 beads from each of the four pools were loaded in the four regions of a PTP fitted with a 4-lane gasket. Sequencing was performed on a GS FLX Titanium System (454 Life Sciences -A Roche Company, Branford). Resulting sequence reads were aligned to HIV-1_HXB2_ by the GS Amplicon Variant Analyzer (AVA) v2.3 software included with the 454 Life Sciences instrument. The AVA software demultiplexed barcoded reads and aligned them to the HIV-1_HXB2_ reference while simultaneously eliminating primer-derived sequences. The software then reported the prevalence of both sequence conservation and mutations relative to HIV-1_HXB2_. Since AVA detects variants in nucleotide space, configuration files were provided to each site that defined amino acid mutations based on triplets of nucleotides. Sff files are deposited in the European Nucleotide Archive (accession number PRJEB12165)

### Data analyses

Data was analyzed for intra- and inter-laboratory concordance using the R statistical package [13] or GraphPad Prism software (GraphPad Software, San Diego, CA). The correlation of variant prevalence for sample replicates within sites and common samples across sites was also computed. In addition, the minimum, maximum and median variant levels, as reported by each site, were calculated to assess concordance with population sequencing results for all samples. For minority variant calling, 1% was chosen as cut off based on previous reports showing combined PCR and pyrosequencing error rates of 0.1–0.3% using HIV-derived clonal sequences [14–16].

Tropism analysis was performed with the geno2pheno co-receptor algorithm for each V3 sample at a false positive rate setting of 3.75% using AVA preprocessed files. Finally, standard population sequencing was performed on each sample using the ViroSeq HIV-1 genotyping system (Abbott Laboratories, Abbott Park, Illinois).

## Results

### Overall performance

Ten out of eleven sites delivered a complete data set. Sequencing information was obtained for 2,986 of 3,520 (85%) expected amplicons. One site was unable to deliver information for the second of their four plates (Table 1) and other sites encountered sporadic amplification failures. Table C in S1 File describes the details for each amplicon drop-out. The higher rate of drop-outs in plate 4 are due to the lower viral titers associated with the dilution series samples. Fig B I in S1 File shows the uniform distribution of the total number of high quality (HQ) reads (those reads that passed the amplicon data processing pipeline) per sequencing region for each site. Furthermore, almost all of the HQ reads could be aligned to HIV-1_HXB2_ with the exception of those from site 27 (region 2) and sites 26 and 28 which delivered a reduced total number of HQ reads.

**Table 1:** Detection across sites of 10 preselected variants. The table lists the percentage of reads carrying the codon variant.

Drug resistance mutation	Sample	HIV-1 subtype	Study sites												
			20	21	23	24	25	26	27	28	29	30	31	Median	IQR
**NNRTI-G190A**	1	G	28.5	23.4	26.5	27.7	27.2	35.3	32.6	18.2	33.0	27.8	27.8	27.8	3.7
**NNRTI-F227L**	3	B	0.0	0.0	0.0	0.0	0.0	0.0	0.0	0.0	0.0	0.0	0.0	0.0	0.0
**NRTI-K219R**	4	B	62.6	58.3	65.6	60.6	58.6	58.2	62.1	67.0	57.2	62.8	65.0	62.1	5.5
**NRTI-V75A**	5	A2	2.8	1.6	1.1	1.1	0.7	1.7	0.7	2.5	0.5	1.2	1.3	1.2	0.8
**PI-p-V82I**	19	B	1.7	1.4	n.a.^a^	1.8	1.1	0.6	—^b^	1.4	0.9	1.4	1.3	1.4	0.3
**PI-I54V**	20	B	54.8	99.3	98.6	98.7	98.4	99.7	0.0	95.2	51.0	49.6	99.2	98.4	46.1
**NRTI-L74I**	21	B	8.7	12.2	15.7	11.4	9.1	11.0	—	10.4	9.7	11.1	11.0	11.0	1.5
**NNRTI-V179D**	24	AG	0.0	0.0	0.0	0.0	0.0	0.0	—	0.0	0.0	0.0	0.0	0.0	0.0
**PI-G73S**	26	B	2.8	3.7	4.5	4.8	4.7	4.9	—	4.1	3.4	1.5	3.6	3.9	1.2
**NRTI-V118I**	27	C	30.0	35.4	32.7	34.8	36.6	56.3	—	39.0	15.1	28.2	27.8	33.8	7.7

^a^ not applicable, i.e., fewer than 15 reads in each direction

^b^ no data returned.

Analysis of the distribution of the 8 amplicons revealed that there was a reduced number of HQ reads (across sites) for amplicons RTP4 and RTP5 for all sites when compared to the number of reads for amplicons RTP1-3, 6, V3A and V3B. Fig B II in S1 File depicts the HQ read distribution data per site for each amplicon using region 3 of the 4-region PTP as a representative example. Across all plates and sites, the median sequencing depth was 1,829 reads per position for RTP (IQR = 592–3,488) and 2,410 reads per position for V3 (IQR = 786–3,695).

### Inter-laboratory concordance

To determine inter-laboratory variability of the current workflow for end users, each site was given a questionnaire to identify and report variant percentages using the AVA software. Ten preselected drug resistance mutations were chosen for this analysis, two were expected negatives (V179D, F227L), three were <5% prevalence (V75A, V82I, G73S) and the remaining five were >10% prevalence (Table 1). All readings reported in this table required a minimum of 15 reads in addition to successful amplicon generation as indicated by the amplification control on the relevant plate. A read coverage of <15 was defined as sequencing failure. Of those amplicons reported with sufficient read coverage, all but one was accurately detected and reported. The single highly inconstant variant was the I54V protease mutation on plate #2 of site 27. The site reported technical errors with this particular plate. Overall, the 10 preselected drug resistant variants, including those with non-B subtypes, showed high inter-laboratory correlation across all sites (Table 1).

### Detection of a minority variant in a plasmid mix

To determine the consistency of variant detection, not only between laboratories but also across plate amplification, an engineered plasmid mixture was sequenced in triplicate at each site (Table 2). The replicate plasmid mixtures were distributed over 3 of the 4 PCR plates and contained a single low level mutation in the protease region, L10R. The mean reported frequencies for this variant *across* all 11 sites for plates 1, 2 and 3 were 4.7, 4.8 and 4.8% with standard deviations of 0.5, 0.6 and 0.3%, respectively. The replicate plates *within* each site had means ranging from 4.3 to 5.2% with standard deviations of 0.3 and 0.4%, respectively (Table 2).

**Table 2:** Consistency of variant detection for an engineered plasmid mix. A plasmid mix containing a variant present at approximately 5% was sequenced in triplicate at each site. The frequency of the minority variant is given in percent. The replicates were distributed over 3 of the 4 PCR plates.

	Plates				
Site	1	2	2	Median	IQR
**20**	4.4	4.0	4.6	4.4	0.3
**21**	5.2	4.7	5.2	5.2	0.3
**23**	5.4	5.1	4.5	5.1	0.5
**24**	4.4	4.3	4.9	4.4	0.3
**25**	4.3	5.1	5.3	5.1	0.5
**26**	4.2	5.0	4.6	4.6	0.4
**27**	4.9	5.9	4.7	4.9	0.6
**28**	4.5	3.7	4.3	4.3	0.4
**29**	4.1	5.2	4.8	4.8	0.6
**30**	5.6	4.7	5.4	5.4	0.4
**31**	5.0	5.1	5.0	5.0	0.0
**Median**	4.5	5.0	4.8		
**IQR**	0.8	0.6	0.5		

### Concordance with population sequencing

The ViroSeq assay was performed on the sample set by a single laboratory to determine concordance with UDS. 99%-100% of drug resistance mutations detected by the population sequencing-based assay were also found by UDS by all sites (Table 3). Three categories of mutations were analyzed: i) major, defined as having a Stanford drug resistance database score >5, ii) polymorphic, defined as having a Stanford drug resistance database score <5 and iii) bulk, defined as any other difference from HIV-1_HXB2_ at the amino acid level. The single discrepancy between UDS and population sequencing for the polymorphic mutation may be due to low sequencing coverage.

**Table 3:** Concordance between amino acid mutations detected by population and ultra-deep pyrosequencing. Included in the analysis were all non-redundant RTP samples (1–9, 19–27, 37 replicate #2, 38 replicate #2, and 39 replicate #2).

Mutation type	Population sequencing	Also detected by 454_UDS (%)
**Major mutations (Stanford score >5)**	72	72 (100)
**Polymorphisms (Stanford score <5)**	113	112 (99)
**Bulk, differences from HIV-1**_**HXB2**_	391	391 (100)

Of clear clinical interest, we found that several population sequencing-called mutations were incorrect due to the inability of population sequencing to phase adjacent nucleotides. Fig C in S1 File is a representative screenshot from the AVA software showing the composition of the UDS sequence reads at codon 215 in the reverse transcriptase. Population sequencing called several possible mutations that could be present at this location although the mutations could not be resolved due to the lack of phase information. This phenomenon occurs since population sequencing only provides information about the composition at each nucleotide location but not about how the nucleotides are connected in each variant. As shown in Fig C in S1 File, UDS resolved the actual variant codon compositions, making it clear that only TAT (Tyr) and ACT (Thr) were present. Population sequencing is not able to disambiguate between the 4 possible combinations TAT (Tyr), AAT (Asn), TCT (Ser), and ACT (Thr) and thus the associated software called all possible known resistance-linked variants at this position. These obvious incorrect combinations in the population sequencing data were excluded prior to the concordance analysis shown in Table 3.

Additional mutations found by UDS but not population sequencing are listed in Table 4. Most of these were minority drug resistance mutations, i.e., found in less than 20% in a given sample. However, several of these mutations were detected at levels >20% by UDS, likely reflecting the inherent variability in population sequencing sensitivity. Most of these mutations were detected and reported at similar frequencies by all sites with sufficient coverage for the respective amplicons (Table 4).

**Table 4:** Drug resistance mutations found only by UDS. For each detected drug resistance mutation, the table indicates the sample, the number of sites detecting the mutation at a frequency above 0.5%, the number of sites with coverage of at least 15 reads and the median, mean, minimum and maximum variant frequencies. Details on reports of each site are given in Table D in S1 File.

Drug resistance mutation	Sample	Sites ≥0.5%^a^	Sites with coverage^b^	Median (%)	Mean (%)	Minimum (%)	Maximum (%)
**NNRTI-F227L**	1	11	11	1.64	1.82	0.83	5.99
**NNRTI-K103N**	2	11	11	10.71	9.98	5.07	15.56
**NNRTI-K103R**	2	9	11	1.23	1.71	0	6.19
**PI-V82A**	3	10	11	4.87	4.55	0	6.37
**NRTI-Y115F**	4	11	11	4.20	3.83	1.14	5.52
**NRTI-K70E**	5	11	11	6.88	6.85	5.21	9.62
**NRTI-V75A**	5	11	11	1.19	1.39	0.55	2.84
**NNRTI-G190A**	7	11	11	3.44	3.33	1.52	5.88
**NNRTI-Y188C**	7	11	11	19.09	19.47	16.29	21.86
**NRTI-M184V**	7	11	11	7.54	7.58	3.22	10.27
**PI-V82A**	7	10	11	2.09	2.06	0.31	3.18
**NNRTI-E138G**	8	5	10	0.66	1.18	0	4.77
**PI-M46V**	8	11	11	2.70	2.79	1.65	4.25
**NNRTI-V179D**	9	11	11	1.43	1.71	1.03	2.74
**NRTI-T215Y**	19	10	10	8.32	8.03	5.55	10.38
**NRTI-T69A**	19	10	10	4.65	5.01	2.97	9.83
**NRTI-T69N**	19	7	10	1.02	1.71	0.16	5.08
**NRTI-M184I**	20	10	10	6.21	6.91	5.12	10.15
**NRTI-L74I**	21	10	10	10.99	11.03	8.77	15.74
**NNRTI-K103R**	23	9	9	19.79	20.82	17.01	26.84
**PI-I47V**	23	9	9	4.30	4.96	2.44	9.87
**NNRTI-E138G**	24	9	10	6.69	7.50	0	17.54
**NNRTI-K103R**	25	9	10	17.97	16.74	0	26.59
**NRTI-K219E**	25	9	10	6.38	5.93	0	9.52
**NRTI-T69N**	25	9	10	22.41	22.38	0	42.57
**NNRTI-F227L**	26	10	10	12.18	12.14	9.31	14.17
**PI-G73S**	26	10	10	3.89	3.81	1.51	4.93

^a^ 0.5% was chosen to cover the expected errors on measurements of a 1% variant

^b^ every site was included reporting at least 15 reads at the certain position. Fewer than 15 reads were defined as unsuccessful amplification.

### Reproducibility and repeatability

**Fig 1:**
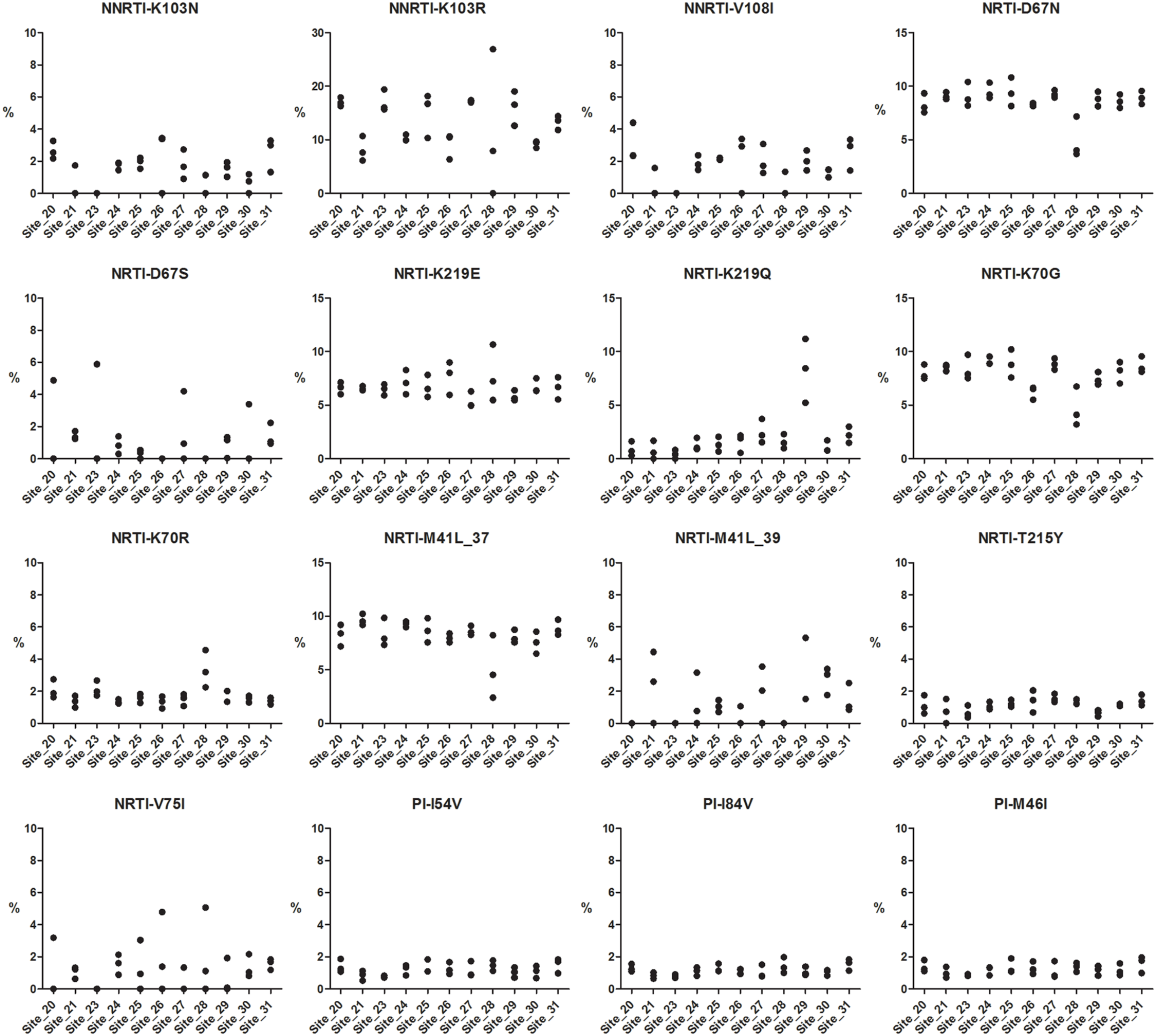
Triplicate sample analysis. A set of three samples was measured in triplicate at each of the 11 sites. Variant percentages measured in the triplicate samples are shown for each mutation.

To determine inter- and intra-laboratory reproducibility, a set of triplicate samples was distributed to each of the sites. The triplicate samples 37, 38 and 39 had previously been sequenced and analyzed in the initial multicenter collaborative study [10]. These samples had viral titers of 230,700, 447,400 and 80,000 copies/mL, respectively. Variant percentages measured in the triplicate samples are shown for each mutation in Fig 1. Triplicate readings for mutations detected in these samples are closely clustered within each site, indicating high intra-site reproducibility (repeatability). The highest degree of variability recorded for several variants was seen in site 28. This increased variation between triplicates may have been related to the very low number of HQ aligned reads in region 3 for this site (Fig B I in S1 File) which was less than half of all other sites. In addition, this data set also indicates high inter-site reproducibility as all mutations were detected across sites with an average standard deviation of 1.0.

### Variant detection in dilution series

**Fig 2:**
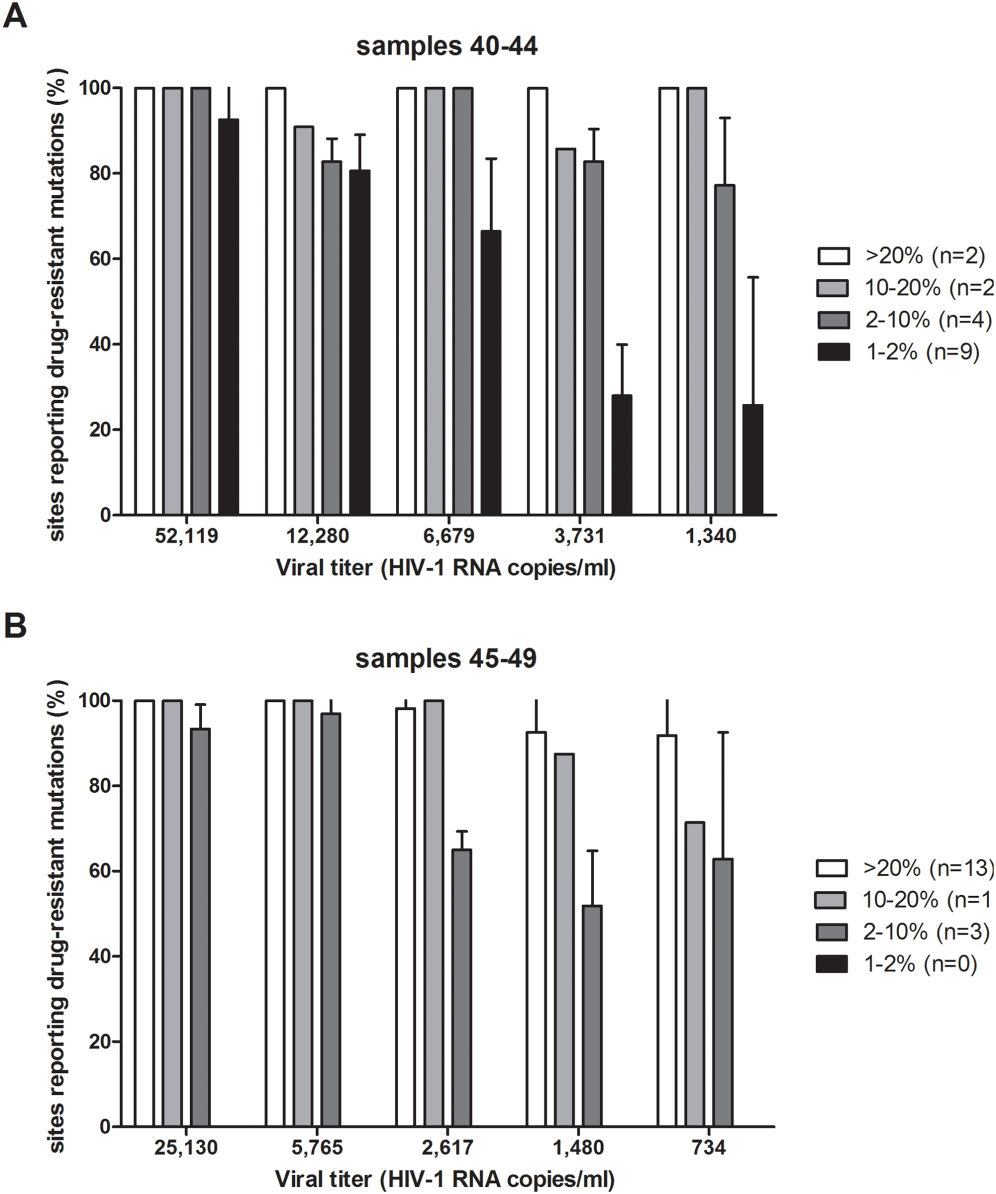
Drug resistance mutation detection in limiting dilution series. Two HIV-1 subtype B samples were used for the dilution series (A, samples 40–44; B, samples 45–49). The four groups shown in the histogram are based on the median frequency of each drug resistance mutation in its respective *undiluted* sample: >20% (white bars), 10–20% (bright grey bars), 2–10% (dark grey bars), and 1–2% (black bars). The means and standard deviations are given of the percentage of sites reporting drug-resistance mutations in these categories. The number of mutations in each category is represented by n.

To examine the limitations of sequencing samples with reduced viral titers, two samples with starting viral titers of 52,119 and 25,130 copies/mL were serially diluted in human plasma four times each to reach a lower viral titer of 1,340 and 734 copies/ml, respectively (Table A in S1 File). To ensure accuracy of the viral titers in the dilution series, the titer was re-determined on aliquots from each dilution. Subsequently, 1 ml of each dilution was extracted, had amplicons generated and was sequenced alongside the other HIV-1 samples. Fig 2 displays the amplicon sequencing coverage for the second dilution series sample. Drug resistance mutations in frequencies of >20% were detected by 90–100% of the sites, independent of the viral titer. Lower viral titers led to a decrease in detection of minority drug resistance mutations, especially when present at 1–2%. Of note, even these very low frequency mutations were still detected (on average) at approximately one quarter of the sites at a viral titer of 1,340 copies/mL (sample 44; Fig 2A).

### Co-receptor usage prediction

Co-receptor usage calls for all V3 samples are shown in Table 5. The geno2pheno_[454]_ algorithm (Max-Planck-Institute for Informatics, Saarbrücken, Germany) was used to analyze the UDS reads with a false positive ratio (FPR) setting of 3.75%. 14 of the samples sequenced by UDS for the V3 region were also analyzed by population sequencing. For those 14 samples, 13 (93%) co-receptor usage calls were concordant between both systems. The single discrepancy between the population sequencing and UDS based co-receptor usage predictions (sample 16) was due to a discordant result from a single site. This site reported 86.4% for V3A and 75.7% for V3B whereas all other sites reported a detection level of 0.0% X4 for both amplicons V3A and V3B and may be due to a sample swap.

**Table 5:** Co-receptor usage predictions. Calculations for UDS were performed using geno2pheno with a false positive rate setting of 3.75% and reported as means with standard deviations for each sample across all sites.

V3 Sample	X4 prediction based on UDS (%)				Prediction based on population sequencing [FPR]^a^
	Amplicon V3-A		Amplicon V3-B		
	Mean	StdDev	Mean	StdDev	
**10**	0.1	0.2	0.6	1.8	R5 [42.3]
**11**	0.0	0.0	0.0	0.0	R5 [12.5]
**12**^b^	n.a.^c^	n.a.	n.a.	n.a.	
**13**	0.0	0.0	0.1	0.4	
**14**	99.8	0.4	99.7	0.3	
**15**	0.0	0.0	0.0	0.0	R5 [93.5]
**16**^d^	6.9	22.8	8.6	27.3	R5 [35.3]
**17**	98.6	4.8	99.2	2.8	
**18**	97.6	2.0	94.8	3.8	X4 [0.2]
**28**	82.8	12.3	84.8	12.5	X4 [0.2]
**29**	86.4	7.5	92.6	4.4	X4 [2.6]
**30**	99.7	0.3	99.2	2.1	X4 [0.1]
**31**	0.2	0.5	0.2	0.6	R5 [35.3]
**32**	0.0	0.1	0.1	0.2	R5 [76.0]
**33**	0.2	0.3	0.3	0.4	R5 [13.8]
**34**	0.1	0.3	0.1	0.2	R5 [38.8]
**35**	0.0	0.0	1.7	5.0	R5 [46.0]
**36**	0.0	0.0	0.0	0.1	R5 [38.5]
**37–2**	99.6	1.1	99.9	0.1	
**38–2**	99.6	1.1	100.0	0.1	
**39–2**	100.0	0.0	100.0	0.0	

^a^ Prediction of HIV-1 tropism by the geno2pheno [co-receptor] algorithm was done using a FPR of 10% according to the European guidelines on the clinical management of HIV-1 tropism testing [34]

^b^ geno2pheno algorithm^3^ only accepted <1% of the reads for sample 12

^c^ not applicable

^d^ The high stdev for sample 16 resulted from a discrepancy at a single site. Without that site the %X4 would have been 0% for both V3A and V3B, agreeing with an R5 population sequencing- based prediction.

## Discussion

The data presented here demonstrate that ultra deep sequencing can provide highly consistent results together with excellent cross-site correlation. For all drug resistance mutations identified by the Stanford Drug Resistance Database in the sequenced region, we demonstrate near perfect concordance with population sequencing results. In addition, 41 mutations were found by UDS in the reverse transcriptase and protease regions at frequencies below 20%. These low frequency mutations could not be detected by population sequencing due to its inherently lower sensitivity [2]. Several studies have previously reported high concordance of UDS and standard population sequencing for mutations present at levels greater than 20–25% (reviewed in [6, 17]). One such study reported similar reproducibility of the UDS results with a correlation value of r^2^ = 0.969 [18].

Additionally, some variants called by population sequencing were incorrect due to the inability of population sequencing to phase individual nucleotides. In the example shown in Fig C in S1 File, population sequencing analysis indicated four possible mutations at codon 215, i.e., T215 (ACT), T215Y (TAT), T215S (TCT), and T215N (AAT). In this particular case, the Serine and Asparagine codons were listed as a combinatorial possibility by the ViroSeq data analysis software, but UDS shows that both are not present among the actual quasispecies. Since T215Y is detected with both methods, it makes no clinical difference in this case, but it is easy to see how in certain instances this could cause interpretation of resistance where none is present, leading to unnecessary avoidance of a potentially useful drug regimen. Looking at the technical performance of the assay, it was clear that certain amplicons performed more robustly than others, both in PCR and sequencing. In general, shorter amplicons can be expected to amplify with higher efficiency than longer ones [19]. This phenomenon negatively affected the number of HQ reads for amplicons RTP4 and RTP5, the two longest amplicons in the set, across all sites. It was also noted that the sequencing run at site 29 yielded a disproportionally large number of HQ reads. This difference was found to be due to a custom change in their software pipeline settings allowing more HQ reads to be reported; however, this can lead to reduced accuracy and is generally not recommended. The AVA software automatically discarded the majority of the additional low accuracy reads and the variant percentages reported from site 29 were similar to those reported by other sites. Conversely, sites 26 and 28 had average read counts but produced a significantly reduced number of aligned reads. This was likely due to the presence of increased amounts of primer dimers that were introduced, along with the amplicons of interest, into the emPCR step prior to the sequencing run. The presence of primer dimers in emPCR is deleterious to the amplification of target amplicons as these shorter fragments are preferentially amplified on the beads [20]. This phenomenon results in a decreased number of amplified target amplicons available for sequencing.

Assay performance at low viral titers was investigated with two dilution series. Data gathered across all sites demonstrated that, even at low titers, mutations present at >20% of the viral population were generally detected. Minority HIV-1 drug resistance mutations in the range of 2–10% were detected at the majority of sites, and minority HIV-1 drug resistance mutations in the range of 1–2% were still detected by some sites. It should be noted that the frequency categories in Fig 2 are based on the variant percentage measured in the undiluted sample which most closely reflects the true representation of the viral species present in the sample. When the viral titer decreases, stochastic sampling and decreased PCR efficiency will cause distortion of the ratio between the different viral species after amplification, and thus the frequencies of individual variants [21].

Sequencing of the V3 region yielded reads spanning the entire V3 loop, which means that each variant can be analyzed without making any inferences about phase. The geno2pheno co-receptor usage prediction software can then simply assign each read to the appropriate predicted tropism group (ie., X4 or R5) and make an overall call based on a relevant false positive rate setting [7, 22]. As noted in the introduction, this approach has been tested in retrospective clinical trials. Theoretically, the increased sensitivity as well as the full phase information should allow for more accurate X4 detection than population based sequencing. Such detailed information is also of great interest for studies of HIV variant dynamics [23, 24]. In this study, the only available comparison data was generated by population sequencing, and we demonstrate good concordance between the two technologies. The single discrepancy noted in Table 5 between the population sequencing and the 454 UDS X4/R5 prediction is for sample 16 where the high standard deviation was caused by an outlying result from a single site. If the data from this site is excluded, the %X4 reporting is 0% for both V3A and V3B amplicons in concordance with the population sequencing based prediction.

There is an increasing awareness of errors that are artificially introduced during RT-PCR and subsequent pyrosequencing and can be falsely interpreted as real mutations [6]. Errors introduced during the cDNA synthesis can currently not be distinguished from real mutations and occur sporadically and randomly [25]. They would need to be the subject of resampling during the following PCR to account for at least 1% of the viral population; the cut-off chosen for our assay. Substitutions occurring during PCR could be addressed using, for instance, random sequence primer identifiers (ID) [26]. Again, our approach to set the cut-off for minority HIV-1 drug-resistance mutations to 1% minimizes false positive calling of most of PCR-introduced mutations except those which occur in the first PCR cycles. Another source of potential error is artificial recombination, the rate of which is highly correlated with the number of DNA templates used in the PCR reaction [14, 27, 28]. The current assay does not contain a step to normalize the transferred cDNA molecules and may therefore not be suitable to link certain drug resistance mutations at low levels, especially in very high viral load samples.

Finally, we must consider errors introduced during the process of pyrosequencing. The 1% cut-off for minority HIV-1 drug-resistance mutations is approximately a magnitude higher than the reported substitution rates introduced by pyrosequencing [29, 30]. In addition, mutations must be detected in both forward and reverse reads at the approximate same frequency to be deemed real. Insertions and deletions (indels) occur predominantly in homopolymer regions [29, 30] and artifactual variant calling can be mitigated by using appropriate platform specific analysis software such as AVA to appropriately align reads and by manually inspecting the alignments in regions with homopolymer runs. Given the very high reproducibility of variants across sites in this study, we feel confident that we have adequately addressed these sources of error for potential use in a clinical setting. In addition, we have greatly reduced the possibilities that exist for introducing human error during manual handling in the laboratory. Compared to the initial multicenter study [10], where several severe errors occurred, the new concept of providing dried down primers in plates proved very successful. In addition to “locking down” the positions of cDNA and PCR primers, we introduced a tracking MID on each plate to ensure that pools from each plate ended up in the intended region on the sequencing run. Additional improvements in the making are color coding and orientation marks on individual plates. As long as materials for individual sequencing runs are kept separate, the main source of manual error remains potential sample swaps, a problem that all clinical laboratories face and have learned to minimize.

454 ultra deep sequencing will only be supported through 2016. Nevertheless, this assay design, either directly or via primer recombination, could be transferred to other next generation sequencing platforms allowing long amplicon sequencing provided by, for instance, Illumina [31, 32], Pacific Biosciences [32], Ion Torrent [32], or Oxford Nanopore Technologies. Regardless of the technology used, our inter- and intra-laboratory testing in a multicenter collaborative study shows that this kind of validation is of the utmost importance when UDS assays are to be considered for implementation in a routine diagnostic setting.

## Conclusion

The field of virology continues to study minority HIV-1 drug resistance mutations and their relevance in the drug resistance field, although the clinical impact is still controversially discussed [8, 9, 33] and there remain challenges such as the search for clinically relevant cut offs. Nevertheless, as next-generation sequencing technologies become more affordable and accessible, they will more regularly play a key role in these studies due to their accuracy, reproducibility and throughput. Thus, intra- and inter-laboratory testing is important for implementation of ultra deep sequencing assays in clinical laboratories for routine diagnostics. The updated assay design described herein for resistance testing and co-receptor usage prediction by ultra deep sequencing allows for an accurate and highly reproducible approach to analysis of the HIV-1 mutant spectra, even at frequencies well below those detected by standard population sequencing.

## Supporting Information

S1 File **Fig A: Overview of amplicon locations.** Reference amplicon sizes (incl. adapters and MIDs) are as follows: (A) RTP1 = 419 bp, RTP2 = 510 bp, RTP3 = 400 bp, RTP4 = 599 bp, RTP5 = 558 bp, RTP6 = 434 bp; (B) V3A = 465 bp, V3B = 423 bp. **Fig B: HQ reads and aligned reads distribution.** I) The number of HQ reads, aligned and not aligned, per sequencing region of the PTP for each site II) The percent composition of total sequencing reads (in brackets below the site name) obtained from each amplicon at each site using region 3 as a representative example. **Fig C: AVA software screen shot of the global alignment of sample 3 from site 31 of low frequency mutation RT215.** Population sequencing indicated the possibility of amino acids Threonine (ACT), Tyrosine (TAT), Serine (TCT), and Asparagine (AAT) at codon 215 in the reverse transcriptase. These could not be further resolved, as the codon nucleotides cannot be phased. The 454 pyrosequencing reads resolved the actual variant codon compositions: 52.0% TAT (Tyr), and 46.9% ACT (Thr) (only mutations present at >1% included). The variants TCT (Ser) and AAT (Asn) could not be detected. **Table A: Overview of sample viral titers and clades used for this study.** 30 HIV-1 subtype B and 6 subtype non-B samples were used for RTP and V3 sequencing, three HIV-1 subtype B samples were run in triplicate (samples 37, 38 and 39) and two HIV-1 subtype B samples were used for the dilution series (samples 40–44 and 45–49). The RTP, dilution samples and triplicate samples are cultured recombinant virus, whereas V3 samples are cultured samples from patient isolates. The viral titer for each is shown. **Table B: Primers. Table C: Details of amplicon drop out across sites per amplicon.** Lack of amplicon production was reported by each site as any amplicon measuring less than 1 ng/μL or that was comprised of primer dimer alone. All amplicon concentration measurements were taken following PCR and purification. **Table D: Drug resistance mutations found only by UDS.** Details on reports of each site. The summary of the data are given in table 4. Hits, number of reads reporting the variant; denom, number of total reads at this position.
(DOC)

## References

[1] Hirsch MS, Gunthard HF, Schapiro JM, Brun-Vezinet F, Clotet B, Hammer SM, et al Antiretroviral drug resistance testing in adult HIV-1 infection: 2008 recommendations of an International AIDS Society-USA panel. Clin Infect Dis. 2008;47(2):266–85. Epub 2008/06/14. 10.1086/589297 . 18549313

[2] Schuurman R, Demeter L, Reichelderfer P, Tijnagel J, de Groot T, Boucher C. Worldwide evaluation of DNA sequencing approaches for identification of drug resistance mutations in the human immunodeficiency virus type 1 reverse transcriptase. J Clin Microbiol. 1999;37(7):2291–6. Epub 1999/06/12. 10364600 10.1128/jcm.37.7.2291-2296.1999 PMC85140

[3] Halvas EK, Aldrovandi GM, Balfe P, Beck IA, Boltz VF, Coffin JM, et al Blinded, multicenter comparison of methods to detect a drug-resistant mutant of human immunodeficiency virus type 1 at low frequency. J Clin Microbiol. 2006;44(7):2612–4. Epub 2006/07/11. 44/7/2612 [pii] 10.1128/JCM.00449-06 16825395 PMC1489464

[4] Paredes R, Marconi VC, Campbell TB, Kuritzkes DR. Systematic evaluation of allele-specific real-time PCR for the detection of minor HIV-1 variants with pol and env resistance mutations. J Virol Methods. 2007;146(1–2):136–46. Epub 2007/07/31. S0166-0934(07)00241-8 [pii] 10.1016/j.jviromet.2007.06.012 . 17662474 PMC4195598

[5] Metzner KJ, Bonhoeffer S, Fischer M, Karanicolas R, Allers K, Joos B, et al Emergence of minor populations of human immunodeficiency virus type 1 carrying the M184V and L90M mutations in subjects undergoing structured treatment interruptions. J Infect Dis. 2003;188(10):1433–43. Epub 2003/11/19. JID30769 [pii] 10.1086/379215 . 14624368

[6] Beerenwinkel N, Gunthard HF, Roth V, Metzner KJ. Challenges and opportunities in estimating viral genetic diversity from next-generation sequencing data. Front Microbiol. 2012;3:329 Epub 2012/09/14. 10.3389/fmicb.2012.00329 22973268 PMC3438994

[7] Swenson LC, Daumer M, Paredes R. Next-generation sequencing to assess HIV tropism. Curr Opin HIV AIDS. 2012;7(5):478–85. Epub 2012/07/27. 10.1097/COH.0b013e328356e9da . 22832710

[8] Li JZ, Paredes R, Ribaudo HJ, Svarovskaia ES, Metzner KJ, Kozal MJ, et al Low-frequency HIV-1 drug resistance mutations and risk of NNRTI-based antiretroviral treatment failure: a systematic review and pooled analysis. JAMA. 2011;305(13):1327–35. Epub 2011/04/07. 305/13/1327 [pii] 10.1001/jama.2011.375 . 21467286 PMC3325645

[9] Cozzi-Lepri A, Noguera-Julian M, Di Giallonardo F, Schuurman R, Daumer M, Aitken S, et al Low-frequency drug-resistant HIV-1 and risk of virological failure to first-line NNRTI-based ART: a multicohort European case-control study using centralized ultrasensitive 454 pyrosequencing. J Antimicrob Chemother. 2015;70(3):930–40. 10.1093/jac/dku426 . 25336166 PMC4319483

[10] Simen BB, Braverman MS, Abbate I, Aerssens J, Bidet Y, Bouchez O, et al An international multicenter study on HIV-1 drug resistance testing by 454 ultra-deep pyrosequencing. J Virol Methods. 2014;204:31–7. 10.1016/j.jviromet.2014.04.007 . 24731928

[11] Shafer RW. Rationale and uses of a public HIV drug-resistance database. J Infect Dis. 2006;194 Suppl 1:S51–8. Epub 2006/08/22. JID35371 [pii] 10.1086/505356 16921473 PMC2614864

[12] Condra JH, Schleif WA, Blahy OM, Gabryelski LJ, Graham DJ, Quintero JC, et al In vivo emergence of HIV-1 variants resistant to multiple protease inhibitors. Nature. 1995;374(6522):569–71. Epub 1995/04/06. 10.1038/374569a0 . 7700387

[13] Team RC. R: A Language and Environment for Statistical Computing. Vienna, Austria 2012 Available from: http://www.R-project.org.

[14] Di Giallonardo F, Zagordi O, Duport Y, Leemann C, Joos B, Kunzli-Gontarczyk M, et al Next-Generation Sequencing of HIV-1 RNA Genomes: Determination of Error Rates and Minimizing Artificial Recombination. PLoS ONE. 2013;8(9):e74249 Epub 2013/09/24. doi: 10.1371/journal.pone.0074249 PONE-D-13-16189 [pii]. . 24058534 10.1371/journal.pone.0074249 PMC3776835

[15] Nicot F, Saliou A, Raymond S, Saune K, Dubois M, Massip P, et al Minority variants associated with resistance to HIV-1 nonnucleoside reverse transcriptase inhibitors during primary infection. J Clin Virol. 2012;55(2):107–13. Epub 2012/07/24. S1386-6532(12)00244-2 [pii] 10.1016/j.jcv.2012.06.018 . 22818969

[16] Varghese V, Shahriar R, Rhee SY, Liu T, Simen BB, Egholm M, et al Minority variants associated with transmitted and acquired HIV-1 nonnucleoside reverse transcriptase inhibitor resistance: implications for the use of second-generation nonnucleoside reverse transcriptase inhibitors. J Acquir Immune Defic Syndr. 2009;52(3):309–15. Epub 2009/09/08. 10.1097/QAI.0b013e3181bca669 . 19734799 PMC2809083

[17] Quinones-Mateu ME, Avila S, Reyes-Teran G, Martinez MA. Deep sequencing: becoming a critical tool in clinical virology. J Clin Virol. 2014;61(1):9–19. 10.1016/j.jcv.2014.06.013 24998424 PMC4119849

[18] Stelzl E, Proll J, Bizon B, Niklas N, Danzer M, Hackl C, et al Human immunodeficiency virus type 1 drug resistance testing: Evaluation of a new ultra-deep sequencing-based protocol and comparison with the TRUGENE HIV-1 Genotyping Kit. J Virol Methods. 2011;178(1–2):94–7. Epub 2011/09/13. S0166-0934(11)00354-5 [pii] 10.1016/j.jviromet.2011.08.020 . 21907239

[19] McCulloch RK, Choong CS, Hurley DM. An evaluation of competitor type and size for use in the determination of mRNA by competitive PCR. PCR Methods Appl. 1995;4(4):219–26. Epub 1995/02/01. . 8574190 10.1101/gr.4.4.219

[20] Vandenbroucke I, Van Marck H, Verhasselt P, Thys K, Mostmans W, Dumont S, et al Minor variant detection in amplicons using 454 massive parallel pyrosequencing: experiences and considerations for successful applications. Biotechniques. 2011;51(3):167–77. Epub 2011/09/13. 000113733 [pii] 10.2144/000113733 . 21906038

[21] Stenman J, Lintula S, Rissanen O, Finne P, Hedstrom J, Palotie A, et al Quantitative detection of low-copy-number mRNAs differing at single nucleotide positions. BioTechniques. 2003;34(1):172–7. Medline:12545556. 12545556 10.2144/03341dd05

[22] Swenson LC, Mo T, Dong WW, Zhong X, Woods CK, Thielen A, et al Deep V3 sequencing for HIV type 1 tropism in treatment-naive patients: a reanalysis of the MERIT trial of maraviroc. Clin Infect Dis. 2011;53(7):732–42. Epub 2011/09/06. cir493 [pii] 10.1093/cid/cir493 . 21890778

[23] Abbate I, Rozera G, Tommasi C, Bruselles A, Bartolini B, Chillemi G, et al Analysis of co-receptor usage of circulating viral and proviral HIV genome quasispecies by ultra-deep pyrosequencing in patients who are candidates for CCR5 antagonist treatment. Clin Microbiol Infect. 2010;17(5):725–31. Epub 2010/08/25. CLM3350 [pii] 10.1111/j.1469-0691.2010.03350.x . 20731681

[24] Rozera G, Abbate I, Bruselles A, Vlassi C, D'Offizi G, Narciso P, et al Massively parallel pyrosequencing highlights minority variants in the HIV-1 env quasispecies deriving from lymphomonocyte sub-populations. Retrovirology. 2009;6:15 10.1186/1742-4690-6-15 19216757 PMC2660291

[25] Roberts JD, Bebenek K, Kunkel TA. The accuracy of reverse transcriptase from HIV-1. Science. 1988;242(4882):1171–3. Epub 1988/11/25. . 2460925 10.1126/science.2460925

[26] Jabara CB, Jones CD, Roach J, Anderson JA, Swanstrom R. Accurate sampling and deep sequencing of the HIV-1 protease gene using a Primer ID. Proc Natl Acad Sci U S A. 2011;108(50):20166–71. Epub 2011/12/03. 1110064108 [pii] 10.1073/pnas.1110064108 . 22135472 PMC3250168

[27] Gorzer I, Guelly C, Trajanoski S, Puchhammer-Stockl E. The impact of PCR-generated recombination on diversity estimation of mixed viral populations by deep sequencing. J Virol Methods. 2010;169(1):248–52. Epub 2010/08/10. S0166-0934(10)00282-X [pii] 10.1016/j.jviromet.2010.07.040 . 20691210

[28] Mild M, Hedskog C, Jernberg J, Albert J. Performance of Ultra-Deep Pyrosequencing in Analysis of HIV-1 pol Gene Variation. PLoS ONE. 2011;6(7):e22741 Epub 2011/07/30. 10.1371/journal.pone.0022741 PONE-D-11-01646 [pii]. 21799940 PMC3143174

[29] Gilles A, Meglecz E, Pech N, Ferreira S, Malausa T, Martin JF. Accuracy and quality assessment of 454 GS-FLX Titanium pyrosequencing. BMC Genomics. 2011;12:245 Epub 2011/05/20. 1471-2164-12-245 [pii] 10.1186/1471-2164-12-245 21592414 PMC3116506

[30] Margulies M, Egholm M, Altman WE, Attiya S, Bader JS, Bemben LA, et al Genome sequencing in microfabricated high-density picolitre reactors. Nature. 2005;437(7057):376–80. 16056220 10.1038/nature03959 PMC1464427

[31] Dudley DM, Bailey AL, Mehta SH, Hughes AL, Kirk GD, Westergaard RP, et al Cross-clade simultaneous HIV drug resistance genotyping for reverse transcriptase, protease, and integrase inhibitor mutations by Illumina MiSeq. Retrovirology. 2014;11:122 10.1186/s12977-014-0122-8 25533166 PMC4302432

[32] Archer J, Weber J, Henry K, Winner D, Gibson R, Lee L, et al Use of Four Next-Generation Sequencing Platforms to Determine HIV-1 Coreceptor Tropism. PLoS ONE. 2012;7(11):e49602 Epub 2012/11/21. 10.1371/journal.pone.0049602 PONE-D-12-18842 [pii]. 23166726 PMC3498215

[33] Metzner KJ, Scherrer AU, Von Wyl V, Boni J, Yerly S, Klimkait T, et al Limited clinical benefit of minority K103N and Y181C-variant detection in addition to routine genotypic resistance testing in antiretroviral therapy-naive patients. AIDS. 2014;28(15):2231–9. 10.1097/QAD.0000000000000397 . 25036184

[34] Vandekerckhove LP, Wensing AM, Kaiser R, Brun-Vezinet F, Clotet B, De Luca A, et al European guidelines on the clinical management of HIV-1 tropism testing. Lancet Infect Dis. 2011;11(5):394–407. Epub 2011/03/25. S1473-3099(10)70319-4 [pii] 10.1016/S1473-3099(10)70319-4 . 21429803

