# Supplementary material for: A Follow-Up of the Multicenter Collaborative Study on HIV-1 Drug Resistance and Tropism Testing Using 454 Ultra Deep Pyrosequencing
Source: PLoS One. 2016 Jan 12;11(1):e0146687. doi: 10.1371/journal.pone.0146687 (PMC4710461; doi:10.1371/journal.pone.0146687)
Supplement: S1 File — Fig A: Overview of amplicon locations. Reference amplicon sizes (incl. adapters and MIDs) are as follows: (A) RTP1 = 419 bp, RTP2 = 510 bp, RTP3 = 400 bp, RTP4 = 599 bp, RTP5 = 558 bp, RTP6 = 434 bp; (B) V3A = 465 bp, V3B = 423 bp. Fig B: HQ reads and aligned reads distribution. I) The number of HQ reads, aligned and not aligned, per sequencing region of the PTP for each site II) The percent composition of total sequencing reads (in brackets below the site name) obtained from each amplicon at each site using region 3 as a representative example. Fig C: AVA software screen shot of the global alignment of sample 3 from site 31 of low frequency mutation RT215. Population sequencing indicated the possibility of amino acids Threonine (ACT), Tyrosine (TAT), Serine (TCT), and Asparagine (AAT) at codon 215 in the reverse transcriptase. These could not be further resolved, as the codon nucleotides cannot be phased. The 454 pyrosequencing reads resolved the actual variant codon compositions: 52.0% TAT (Tyr), and 46.9% ACT (Thr) (only mutations present at >1% included). The variants TCT (Ser) and AAT (Asn) could not be detected. Table A: Overview of sample viral titers and clades used for this study. 30 HIV-1 subtype B and 6 subtype non-B samples were used for RTP and V3 sequencing, three HIV-1 subtype B samples were run in triplicate (samples 37, 38 and 39) and two HIV-1 subtype B samples were used for the dilution series (samples 40–44 and 45–49). The RTP, dilution samples and triplicate samples are cultured recombinant virus, whereas V3 samples are cultured samples from patient isolates. The viral titer for each is shown. Table B: Primers. Table C: Details of amplicon drop out across sites per amplicon. Lack of amplicon production was reported by each site as any amplicon measuring less than 1 ng/μL or that was comprised of primer dimer alone. All amplicon concentration measurements were taken following PCR and purification. Table D: Drug resistance mutations found only by [file pone.0146687.s001.doc]

**Supporting information**

**A follow-up of the Multicenter Collaborative Study on HIV-1 Drug Resistance and Tropism Testing using 454 Ultra Deep Pyrosequencing**

Elizabeth P. St. John^1^, Birgitte B. Simen^1^, Gregory S. Turenchalk^1^, Michael S. Braverman^1^, Isabella Abbate^2^, Jeroen Aerssens^3^, Olivier Bouchez^4^, Christian Gabriel^5^, Jacques Izopet^6^, Karolin Meixenberger^7^, Francesca Di Giallonardo^8^, Ralph Schlapbach^9^, Roger Paredes^10^, James Sakwa^11^, Gudrun G. Schmitz-Agheguian^12^, Alexander Thielen^13^, Martin Victor^12^, Karin J. Metzner^8,14,*, ¶^, and Martin P. Däumer^15, ¶^, on behalf of the 454 HIV-1 Alpha Study Group

^1^ 454 Life Sciences, A Roche Company, Branford, CT, USA

^2^ National Institute for Infectious Diseases “L. Spallanzani, Rome, Italy

^3^ Janssen Infectious Diseases - Diagnostics bvba, Beerse, Belgium

^4^ Plateforme Génomique Toulouse/Laboratoire Génétique Cellulaire, Toulouse, France

^5^ Blutzentrale Linz, Linz, Austria

^6^ INSERM U563, Toulouse, France

^7^ Robert Koch-Institute, Berlin, Germany

^8^ Division of Infectious Diseases and Hospital Epidemiology, University Hospital Zurich, University of Zurich, Zurich, Switzerland

^9^ Functional Genomics Center Zurich, University of Zurich, ETH Zurich, Zurich Switzerland

^10^ Institut de Recerca de la SIDA – IrsiCaixa, Badalona, Spain

^11^ Technology Innovation Agency-National Genomics Platform, Durban, South Africa

^12^ Roche Applied Science, Penzberg, Germany

^13^ Max-Planck-Institute for Informatics, Saarbrücken, Germany

^14^ Institute of Medical Virology, University of Zurich, Zurich, Switzerland

^15^ Institute of Immunology and Genetics, Kaiserslautern, Germany

**Figure A: Overview of amplicon locations.** Reference amplicon sizes (incl. adapters and MIDs) are as follows: (A) RTP1=419 bp, RTP2=510 bp, RTP3=400 bp, RTP4=599 bp, RTP5=558 bp, RTP6=434 bp; (B) V3A=465 bp, V3B=423 bp.

A


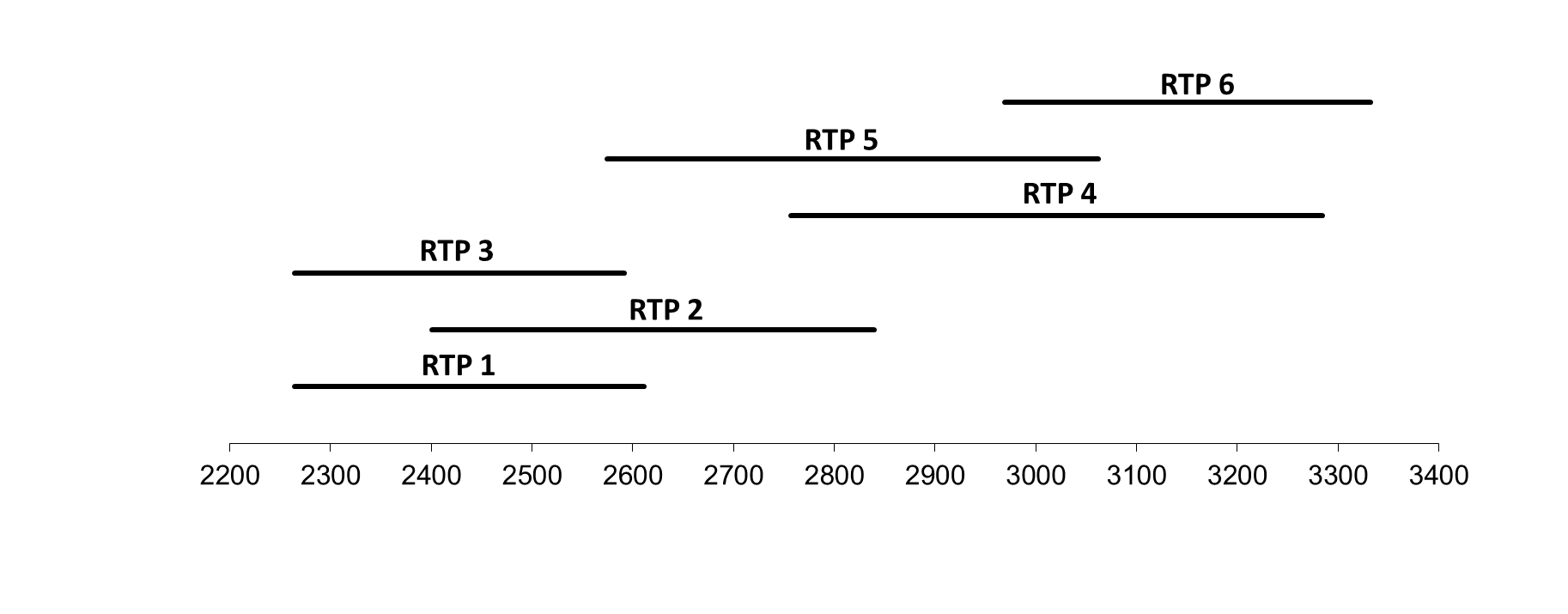


B


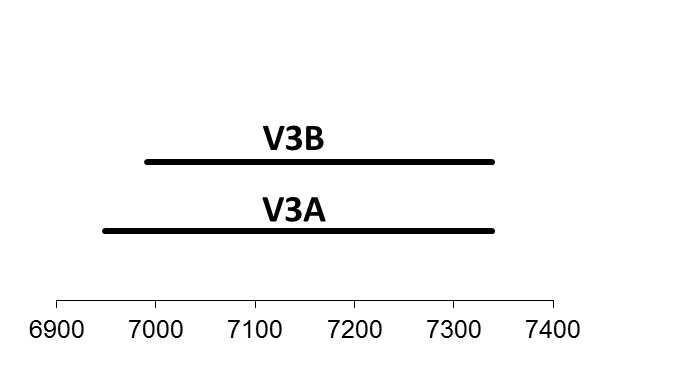


**Figure B: HQ reads and aligned reads distribution.** A) The number of HQ reads, aligned and not aligned, per sequencing region of the PTP for each site B) The percent composition of total sequencing reads (in brackets below the site name) obtained from each amplicon at each site using region 3 as a representative example.


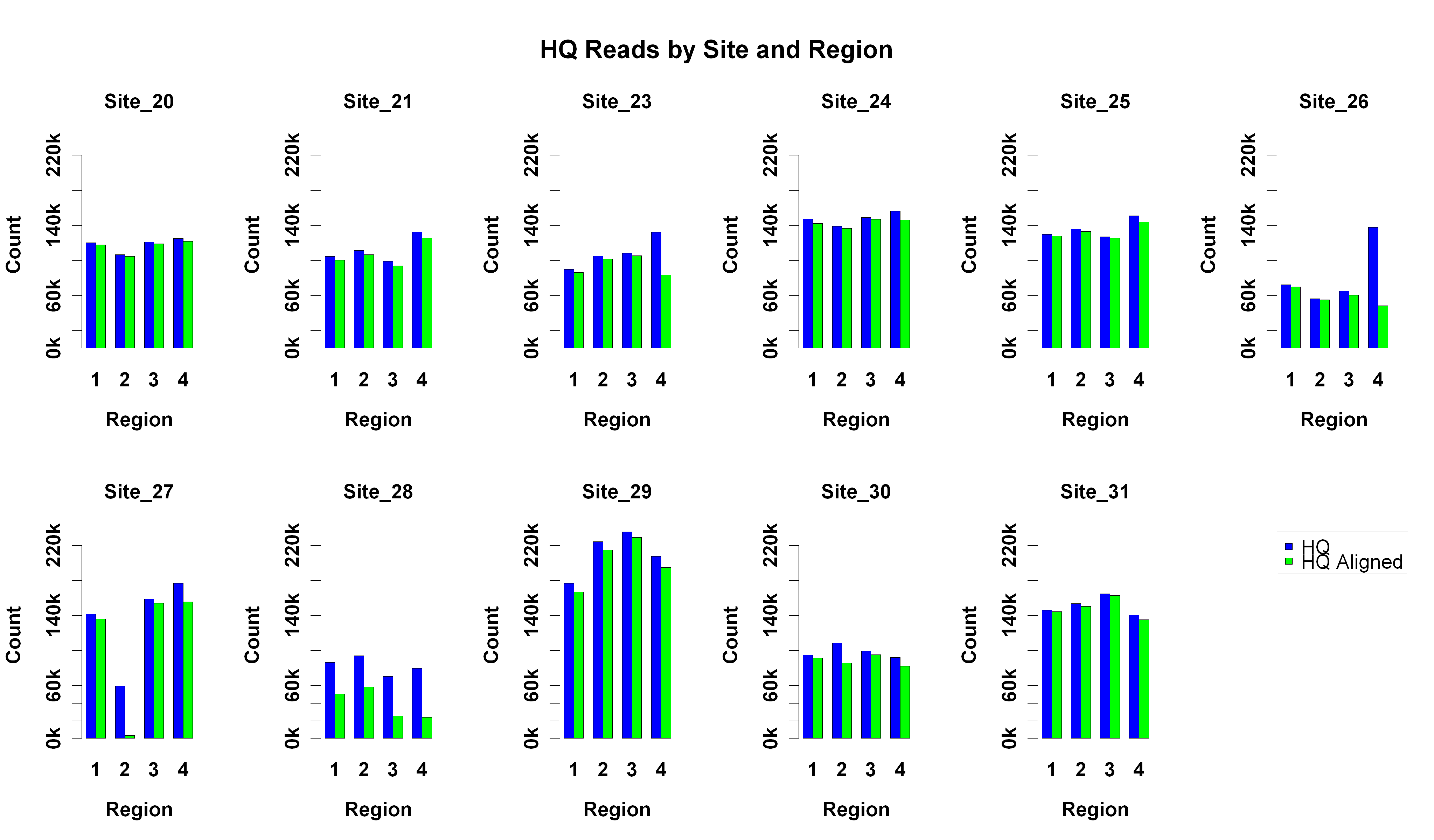


II

I


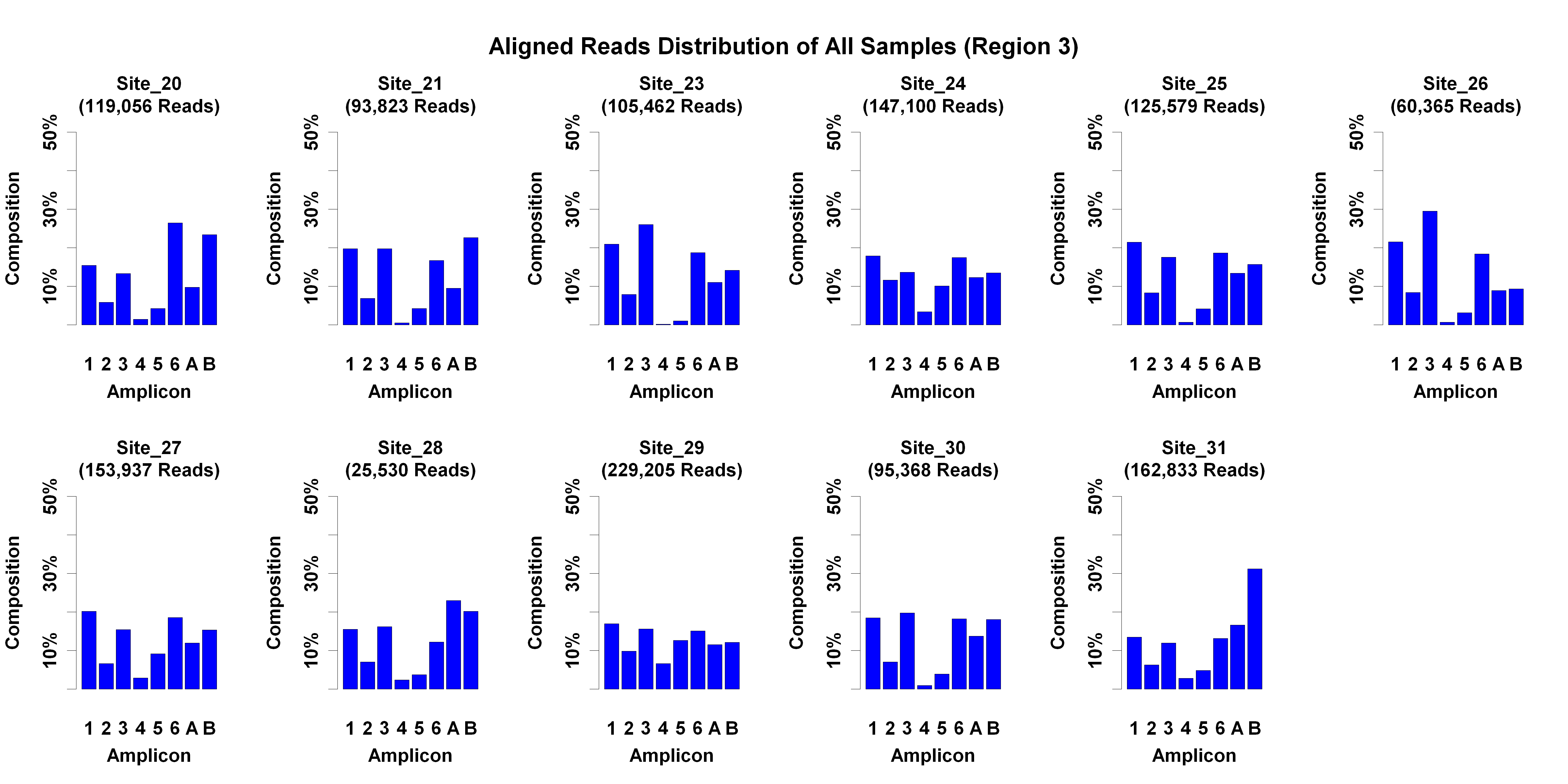


A

**Figure C: AVA software screen shot of the global alignment of sample 3 from site 31 of low frequency mutation RT215.** Population sequencing indicated the possibility of amino acids Threonine (ACT), Tyrosine (TAT), Serine (TCT), and Asparagine (AAT) at codon 215 in the reverse transcriptase. These could not be further resolved, as the codon nucleotides cannot be phased. The 454 pyrosequencing reads resolved the actual variant codon compositions: 52.0% TAT (Tyr), and 46.9% ACT (Thr) (only mutations present at >1% included). The variants TCT (Ser) and AAT (Asn) could not be detected.

**
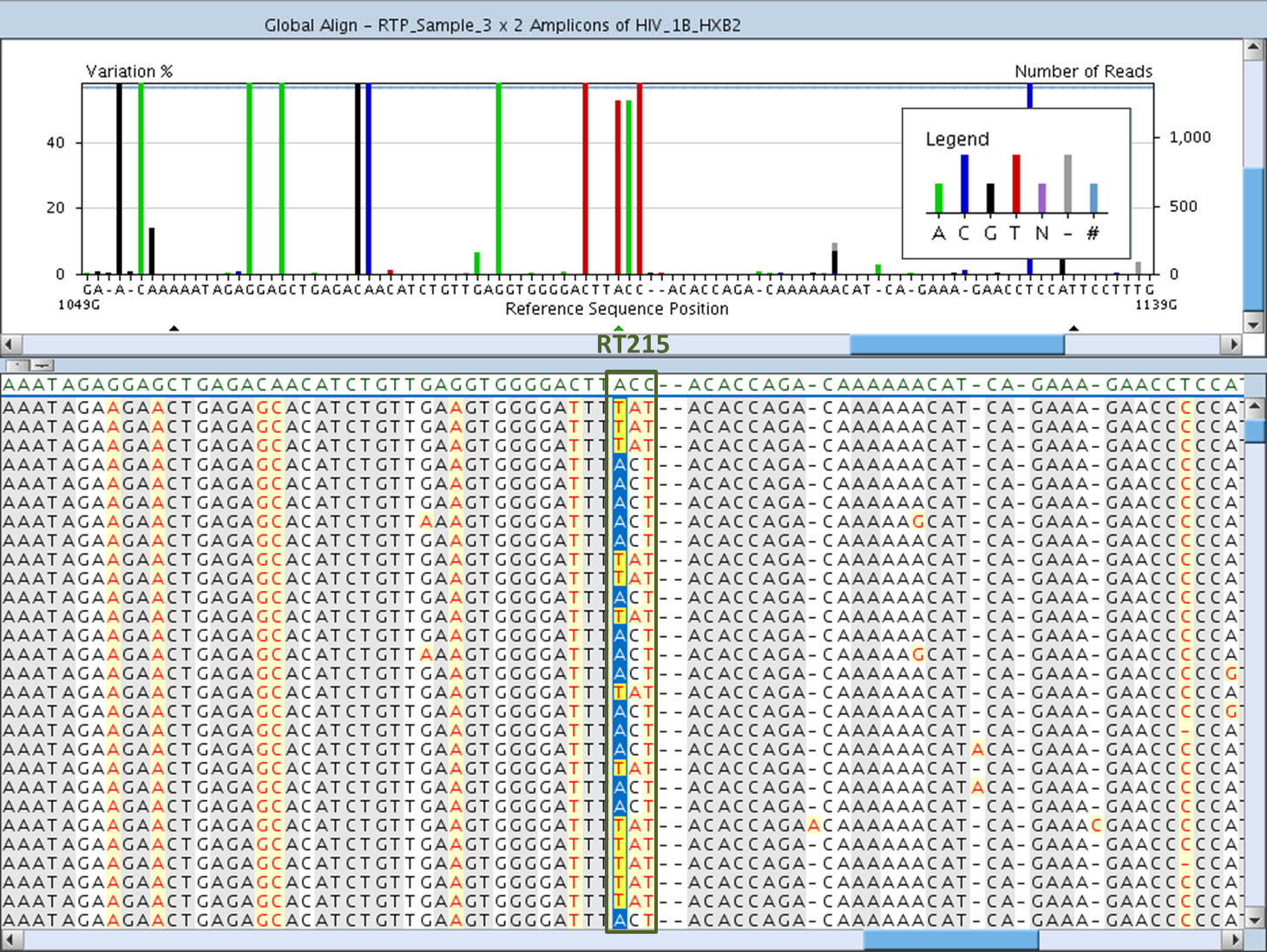
**

**Table A: Overview of sample viral titers and clades used for this study.** 30 HIV-1 subtype B and 6 subtype non-B samples were used for RTP and V3 sequencing, three HIV-1 subtype B samples were run in triplicate (samples 37, 38 and 39) and two HIV-1 subtype B samples were used for the dilution series (samples 40-44 and 45-49). The RTP, dilution samples and triplicate samples are cultured recombinant virus, whereas V3 samples are cultured samples from patient isolates. The viral titer for each is shown.

**Table B**: Primers.

| Amplicon | Primer | Sequence (5’-3’) |
| --- | --- | --- |
| RTP1 | RTP 1F | ATCACTCTTTGGCARCGACC |
|  | RTP 1R | TTGGGCCATCCATTCCTGG |
| RTP2 | RTP 2F | GGAATTGGAGGTTTTATCAARGT |
|  | RTP 2R | TGTGGTATTCCTAATTGAACYTCCCA |
| RTP3 | RTP 3F | ATCACTCTTTGGCARCGACC |
|  | RTP 3R | CTTTAATTTTACTGGTACAGTTTCAAT |
| RTP4 | RTP 4F | TACTAARTGGAGAAAATTAGTAGA |
|  | RTP 4R | TATAGGCTGTACTGTCCATTTRTC |
| RTP5 | RTP 5F | GTACCAGTAAAATTAAAGCCAGGRA |
|  | RTP 5R | GGCTCTAAGATTTTTGTCATGCT |
| RTP6 | RTP 6F | CACCAGGGATTAGATATCAGTACAATGT |
|  | RTP 6R | AACTTCTGTATATCATTGACAGTCCA |
| V3A | V3 AF | TCAGCACAGTACARTGYACACATGG |
|  | V3 AR | CATTACAATTTCTRGGTCYCCTCC |
| V3B | V3 BF | CAACTCAACTRCTGTTAAATGGYAG |
|  | V3 BR | CATTACAATTTCTRGGTCYCCTCC |

**Table C: Details of amplicon drop out across sites per amplicon.** Lack of amplicon production was reported by each site as any amplicon measuring less than 1 ng/μL or that was comprised of primer dimer alone. All amplicon concentration measurements were taken following PCR and purification.

**Table D: Drug resistance mutations found only by UDS.** Details on reports of each site. The summary of the data are given in table 4. Hits, number of reads reporting the variant; denom, number of total reads at this position

| **Sample** | **Variant Name** | **Site_20** | | | **Site_21** | | | **Site_23** | | | **Site_24** | | |
| --- | --- | --- | --- | --- | --- | --- | --- | --- | --- | --- | --- | --- | --- |
|  |  | **Percent** | **Hits** | **Denom** | **Percent** | **Hits** | **Denom** | **Percent** | **Hits** | **Denom** | **Percent** | **Hits** | **Denom** |
| **1** | **NNRTI-F227L** | 1.68% | 40 | 2387 | 0.92% | 27 | 2922 | 1.80% | 34 | 1893 | 0.83% | 26 | 3138 |
| **2** | **NNRTI-K103N** | 6.34% | 73 | 1152 | 12.02% | 109 | 907 | 8.53% | 18 | 211 | 12.90% | 359 | 2784 |
| **2** | **NNRTI-K103R** | 1.22% | 14 | 1152 | 0.99% | 9 | 907 | 0.47% | 1 | 211 | 1.15% | 32 | 2784 |
| **3** | **PI-V82A** | 5.24% | 365 | 6960 | 4.70% | 196 | 4166 | 6.17% | 221 | 3583 | 4.35% | 259 | 5948 |
| **4** | **NRTI-Y115F** | 3.72% | 27 | 725 | 4.58% | 32 | 698 | 2.30% | 4 | 174 | 4.20% | 100 | 2380 |
| **5** | **NRTI-K70E** | 8.15% | 215 | 2639 | 5.58% | 101 | 1809 | 7.85% | 158 | 2012 | 6.97% | 234 | 3355 |
| **5** | **NRTI-V75A** | 2.84% | 75 | 2638 | 1.66% | 30 | 1810 | 1.09% | 22 | 2012 | 1.10% | 37 | 3355 |
| **7** | **NNRTI-G190S** | 3.61% | 75 | 2080 | 1.92% | 43 | 2235 | 1.99% | 22 | 1104 | 2.75% | 75 | 2728 |
| **7** | **NNRTI-Y188C** | 19.09% | 397 | 2080 | 20.84% | 466 | 2236 | 16.29% | 180 | 1105 | 18.52% | 505 | 2727 |
| **7** | **NRTI-M184V** | 6.11% | 127 | 2077 | 9.26% | 207 | 2235 | 6.58% | 73 | 1109 | 9.57% | 261 | 2727 |
| **7** | **PI-V82A** | 2.12% | 176 | 8294 | 2.06% | 79 | 3833 | 3.18% | 129 | 4057 | 2.34% | 166 | 7084 |
| **8** | **NNRTI-E138G** | 0.00% | 0 | 525 | 2.31% | 9 | 389 | 0.00% | 0 | 109 | 1.92% | 19 | 990 |
| **8** | **PI-M46V** | 3.36% | 117 | 3485 | 2.70% | 64 | 2374 | 2.92% | 76 | 2607 | 2.39% | 68 | 2841 |
| **9** | **NNRTI-V179D** | 1.33% | 33 | 2478 | 2.74% | 57 | 2084 | 1.28% | 28 | 2180 | 1.03% | 29 | 2814 |
| **19** | **NRTI-T215Y** | 8.48% | 139 | 1640 | 8.07% | 137 | 1698 | 8.33% | 207 | 2485 | 5.55% | 189 | 3408 |
| **19** | **NRTI-T69A** | 5.17% | 67 | 1296 | 5.37% | 67 | 1248 | 3.19% | 3 | 94 | 2.97% | 87 | 2931 |
| **19** | **NRTI-T69N** | 0.62% | 8 | 1296 | 0.16% | 2 | 1248 | 2.13% | 2 | 94 | 0.41% | 12 | 2931 |
| **20** | **NRTI-M184I** | 6.33% | 155 | 2447 | 5.85% | 129 | 2204 | 8.87% | 182 | 2052 | 5.36% | 191 | 3563 |
| **21** | **NRTI-L74I** | 8.77% | 158 | 1802 | 12.17% | 98 | 805 | 15.74% | 54 | 343 | 11.42% | 364 | 3188 |
| **23** | **NNRTI-K103R** | 21.68% | 204 | 941 | 19.67% | 118 | 600 | 17.01% | 41 | 241 | 18.42% | 336 | 1824 |
| **23** | **PI-I47V** | 5.73% | 206 | 3594 | 3.95% | 139 | 3523 | 3.50% | 185 | 5285 | 4.30% | 145 | 3371 |
| **24** | **NNRTI-E138G** | 6.75% | 42 | 622 | 6.16% | 26 | 422 | 17.54% | 30 | 171 | 5.43% | 86 | 1585 |
| **25** | **NNRTI-K103R** | 26.49% | 182 | 687 | 21.32% | 132 | 619 | 14.12% | 25 | 177 | 19.10% | 267 | 1398 |
| **25** | **NRTI-K219E** | 5.98% | 93 | 1556 | 7.04% | 137 | 1946 | 6.54% | 90 | 1377 | 4.18% | 140 | 3352 |
| **25** | **NRTI-T69N** | 20.08% | 314 | 1564 | 21.38% | 382 | 1787 | 23.38% | 350 | 1497 | 25.65% | 731 | 2850 |
| **26** | **NNRTI-F227L** | 13.65% | 209 | 1531 | 12.50% | 137 | 1096 | 10.77% | 177 | 1644 | 11.84% | 287 | 2423 |
| **26** | **PI-G73S** | 2.85% | 105 | 3689 | 3.71% | 134 | 3608 | 4.54% | 197 | 4340 | 4.78% | 265 | 5545 |

| **Sample** | **Variant Name** | **Site_25** | | | **Site_26** | | | **Site_27** | | | **Site_28** | | |
| --- | --- | --- | --- | --- | --- | --- | --- | --- | --- | --- | --- | --- | --- |
|  |  | **Percent** | **Hits** | **Denom** | **Percent** | **Hits** | **Denom** | **Percent** | **Hits** | **Denom** | **Percent** | **Hits** | **Denom** |
| **1** | **NNRTI-F227L** | 1.64% | 27 | 1647 | 1.69% | 24 | 1420 | 1.96% | 74 | 3780 | 1.64% | 13 | 795 |
| **2** | **NNRTI-K103N** | 11.51% | 110 | 956 | 15.56% | 63 | 405 | 6.88% | 129 | 1876 | 5.07% | 22 | 434 |
| **2** | **NNRTI-K103R** | 1.46% | 14 | 956 | 0.00% | 0 | 405 | 1.23% | 23 | 1876 | 2.30% | 10 | 434 |
| **3** | **PI-V82A** | 5.82% | 376 | 6466 | 4.87% | 155 | 3180 | 3.33% | 200 | 5999 | 6.37% | 148 | 2323 |
| **4** | **NRTI-Y115F** | 5.36% | 29 | 541 | 1.14% | 4 | 352 | 4.87% | 76 | 1562 | 2.30% | 7 | 304 |
| **5** | **NRTI-K70E** | 5.21% | 137 | 2632 | 5.28% | 38 | 720 | 7.58% | 239 | 3152 | 6.88% | 114 | 1656 |
| **5** | **NRTI-V75A** | 0.68% | 18 | 2632 | 1.67% | 12 | 720 | 0.67% | 21 | 3152 | 2.48% | 41 | 1656 |
| **7** | **NNRTI-G190S** | 1.52% | 38 | 2504 | 4.84% | 51 | 1053 | 4.67% | 106 | 2269 | 3.89% | 53 | 1364 |
| **7** | **NNRTI-Y188C** | 18.45% | 463 | 2510 | 20.89% | 220 | 1053 | 19.04% | 432 | 2269 | 21.86% | 299 | 1368 |
| **7** | **NRTI-M184V** | 8.04% | 202 | 2511 | 9.50% | 100 | 1053 | 10.27% | 233 | 2268 | 3.22% | 44 | 1368 |
| **7** | **PI-V82A** | 2.09% | 170 | 8137 | 1.58% | 53 | 3365 | 3.03% | 159 | 5241 | 0.31% | 11 | 3520 |
| **8** | **NNRTI-E138G** | 1.47% | 7 | 475 | 0.00% | 0 | 312 | 1.31% | 13 | 990 | 0.00% | 0 | 421 |
| **8** | **PI-M46V** | 1.79% | 68 | 3797 | 2.73% | 65 | 2383 | 1.65% | 48 | 2917 | 4.25% | 76 | 1787 |
| **9** | **NNRTI-V179D** | 2.31% | 63 | 2726 | 2.36% | 31 | 1316 | 2.53% | 75 | 2962 | 1.43% | 14 | 977 |
| **19** | **NRTI-T215Y** | 8.31% | 167 | 2010 | 10.38% | 246 | 2370 | 0.00% | 0 | 0 | 7.15% | 54 | 755 |
| **19** | **NRTI-T69A** | 3.90% | 74 | 1898 | 5.08% | 56 | 1102 | 0.00% | 0 | 0 | 9.83% | 59 | 600 |
| **19** | **NRTI-T69N** | 0.79% | 15 | 1898 | 5.08% | 56 | 1102 | 0.00% | 0 | 0 | 0.33% | 2 | 600 |
| **20** | **NRTI-M184I** | 5.46% | 132 | 2418 | 10.15% | 304 | 2995 | 0.00% | 0 | 0 | 7.86% | 101 | 1285 |
| **21** | **NRTI-L74I** | 9.06% | 214 | 2361 | 11.02% | 112 | 1016 | 0.00% | 0 | 0 | 10.37% | 161 | 1552 |
| **23** | **NNRTI-K103R** | 20.09% | 224 | 1115 | 0.00% | 0 | 0 | 0.00% | 0 | 0 | 25.60% | 96 | 375 |
| **23** | **PI-I47V** | 5.28% | 221 | 4189 | 0.00% | 0 | 0 | 0.00% | 0 | 0 | 9.87% | 241 | 2441 |
| **24** | **NNRTI-E138G** | 6.68% | 29 | 434 | 3.16% | 6 | 190 | 0.00% | 0 | 0 | 13.56% | 32 | 236 |
| **25** | **NNRTI-K103R** | 9.92% | 83 | 837 | 19.31% | 45 | 233 | 0.00% | 0 | 0 | 26.59% | 96 | 361 |
| **25** | **NRTI-K219E** | 7.49% | 165 | 2204 | 9.52% | 57 | 599 | 0.00% | 0 | 0 | 8.18% | 104 | 1272 |
| **25** | **NRTI-T69N** | 24.36% | 593 | 2434 | 42.57% | 278 | 653 | 0.00% | 0 | 0 | 23.13% | 217 | 938 |
| **26** | **NNRTI-F227L** | 13.44% | 237 | 1763 | 12.85% | 169 | 1315 | 0.00% | 0 | 0 | 14.17% | 122 | 861 |
| **26** | **PI-G73S** | 4.73% | 216 | 4569 | 4.93% | 182 | 3688 | 0.00% | 0 | 0 | 4.06% | 81 | 1996 |

| **Sample** | **Variant Name** | **Site_29** | | | **Site_30** | | | **Site_31** | | |
| --- | --- | --- | --- | --- | --- | --- | --- | --- | --- | --- |
|  |  | **Percent** | **Hits** | **Denom** | **Percent** | **Hits** | **Denom** | **Percent** | **Hits** | **Denom** |
| **1** | **NNRTI-F227L** | 5.99% | 139 | 2320 | 0.88% | 15 | 1713 | 1.01% | 18 | 1781 |
| **2** | **NNRTI-K103N** | 11.67% | 292 | 2503 | 8.56% | 53 | 619 | 10.71% | 132 | 1232 |
| **2** | **NNRTI-K103R** | 6.19% | 155 | 2503 | 1.94% | 12 | 619 | 1.87% | 23 | 1232 |
| **3** | **PI-V82A** | 0.00% | 0 | 6346 | 3.81% | 157 | 4122 | 5.38% | 230 | 4275 |
| **4** | **NRTI-Y115F** | 5.28% | 233 | 4410 | 2.90% | 13 | 448 | 5.52% | 45 | 815 |
| **5** | **NRTI-K70E** | 6.64% | 351 | 5286 | 9.62% | 129 | 1341 | 5.54% | 119 | 2149 |
| **5** | **NRTI-V75A** | 0.55% | 29 | 5282 | 1.19% | 16 | 1341 | 1.35% | 29 | 2149 |
| **7** | **NNRTI-G190S** | 2.07% | 75 | 3625 | 5.88% | 106 | 1804 | 3.44% | 36 | 1047 |
| **7** | **NNRTI-Y188C** | 21.08% | 764 | 3625 | 17.52% | 316 | 1804 | 20.63% | 216 | 1047 |
| **7** | **NRTI-M184V** | 7.54% | 273 | 3623 | 7.36% | 133 | 1806 | 5.90% | 62 | 1050 |
| **7** | **PI-V82A** | 1.50% | 128 | 8509 | 2.85% | 117 | 4099 | 1.57% | 72 | 4587 |
| **8** | **NNRTI-E138G** | 0.00% | 0 | 1603 | 0.00% | 0 | 0 | 4.77% | 23 | 482 |
| **8** | **PI-M46V** | 2.40% | 92 | 3835 | 3.93% | 92 | 2339 | 2.55% | 53 | 2076 |
| **9** | **NNRTI-V179D** | 1.44% | 53 | 3693 | 1.12% | 16 | 1434 | 1.24% | 14 | 1125 |
| **19** | **NRTI-T215Y** | 6.83% | 348 | 5097 | 8.42% | 169 | 2008 | 8.76% | 280 | 3195 |
| **19** | **NRTI-T69A** | 3.83% | 194 | 5059 | 4.21% | 40 | 951 | 6.60% | 196 | 2969 |
| **19** | **NRTI-T69N** | 4.70% | 238 | 5059 | 1.58% | 15 | 951 | 1.25% | 37 | 2969 |
| **20** | **NRTI-M184I** | 8.04% | 590 | 7342 | 5.12% | 192 | 3751 | 6.09% | 100 | 1642 |
| **21** | **NRTI-L74I** | 9.75% | 386 | 3959 | 11.01% | 98 | 890 | 10.97% | 214 | 1951 |
| **23** | **NNRTI-K103R** | 19.79% | 656 | 3315 | 26.84% | 244 | 909 | 18.31% | 215 | 1174 |
| **23** | **PI-I47V** | 3.44% | 143 | 4159 | 6.17% | 245 | 3973 | 2.44% | 53 | 2176 |
| **24** | **NNRTI-E138G** | 9.02% | 515 | 5709 | 0.00% | 0 | 232 | 6.69% | 55 | 822 |
| **25** | **NNRTI-K103R** | 16.83% | 1140 | 6772 | 13.76% | 71 | 516 | 0.00% | 0 | 463 |
| **25** | **NRTI-K219E** | 4.11% | 240 | 5840 | 6.22% | 112 | 1802 | 0.00% | 0 | 1862 |
| **25** | **NRTI-T69N** | 21.58% | 1613 | 7476 | 21.68% | 191 | 881 | 0.00% | 0 | 2364 |
| **26** | **NNRTI-F227L** | 11.86% | 412 | 3475 | 9.31% | 27 | 290 | 11.02% | 147 | 1334 |
| **26** | **PI-G73S** | 3.41% | 172 | 5042 | 1.51% | 32 | 2114 | 3.57% | 109 | 3049 |
